# The impact of allergic rhinitis on the management of asthma in a working population

**DOI:** 10.1186/s12890-015-0136-6

**Published:** 2015-11-09

**Authors:** Dorothée Provost, Yuriko Iwatsubo, Stéphanie Riviere, Maëlaïg Mevel, Alain Didier, Patrick Brochard, Ellen Imbernon, Chantal Raherison

**Affiliations:** Département Santé Travail, Institut de veille sanitaire (InVS), F-94415 Saint-Maurice, France; ISPED, Centre Inserm U897 – Epidémiologie-Biostatistique, Équipe associée en santé travail (Essat), Univ. Bordeaux, F-33076 Bordeaux, France; Service de Pneumologie-Allergologie, CHU de Toulouse, F-31059 Toulouse, France; Service de médecine du travail et pathologies professionnelles, CHU de Bordeaux, F-33076 Bordeaux, France; Service des maladies respiratoires, CHU de Bordeaux, F-33076 Bordeaux, France

**Keywords:** Allergic rhinitis, Asthma, Asthma control, Occupation, Asthma severity

## Abstract

**Background:**

Currently, little data is available about the management of asthma in the working population. The aim of this study was to describe asthma control and severity among workers according to current or previous allergic rhinitis comorbidity.

**Methods:**

A network of occupational physicians participated in this pilot study on a voluntary basis. They included a random sample of salaried workers during their systematic occupational medical check-up. All subjects completed a self-administered questionnaire based on the European Community Respiratory Health Survey screening questionnaire, and if they reported any respiratory symptoms including allergic rhinitis, the physician filled in a medical questionnaire. Current asthma control and severity were evaluated according to 2006 Global Initiative for Asthma guidelines.

**Results:**

A total of 110 occupational physicians from two French regions participated. Out of the 6906 employees screened, 3102 identified respiratory symptoms and completed the medical questionnaire and performed spirometry. Overall, 374 were identified as current asthmatics, including 271 (72.5 %) with allergic rhinitis. Among current asthmatics with current allergic rhinitis (*n* = 95), 68.8 % had partially controlled asthma or uncontrolled asthma, including 51.6 % who received insufficient anti-asthmatic treatment. Partly or no control asthma was not associated with current rhinitis (OR = 1.4; 95 % CI: 0.8-2.7). Current asthmatics with current or previous allergic rhinitis had a significantly lower risk of emergency department visits than current asthmatics without allergic rhinitis (respectively 11.6, 17.1 and 29.1 %; *P* = 0.002).

**Conclusions:**

Most current asthmatics both with and without allergic rhinitis had uncontrolled asthma, with inappropriate treatment. Future intervention strategies need to be developed for effective control and prevention of asthma in the workplace.

## Background

Asthma is a chronic disease characterized by acute symptomatic episodes of varying severity that can, in severe cases, be near-fatal or fatal. It affects around 6 % of the general adult population in France [1]. Allergic rhinitis (AR) is three times more prevalent than asthma [2] and tends to occur three times more frequently in occupational settings than in settings outside the workplace [3]. A recent population-based survey indicated that approximately 15 % of adult-onset asthma could be attributable to the workplace environment [4]. Reports on time trends in atopic disease suggest that the prevalence of asthma and AR has leveled-off in industrialized countries after several decades of increase [5]. This apparent change for the better could be due to the implementation of the Global Initiative for Asthma (GINA) guidelines which have been introduced to improve patient care and provide optimal long-term asthma control [6, 7]. However, recent surveys conducted in Europe and the United States have indicated that control of asthma remains suboptimal; many patients continue to have frequent symptoms and asthma exacerbations, as well as limitations on their daily activities [8].

AR is a common co-morbid condition associated with asthma [9] and the majority of patients with asthma suffer from AR [10]. AR is also reported as a predictive factor of future asthma [10, 11]. The International Primary Care Respiratory Group has suggested an approach to control asthma based on identifying clinical and behavioral factors associated with poor control, with AR included as a clinical factor [12]. Clinically diagnosed AR is associated with significantly worse asthma control in adults [13, 14]. However, very little data is currently available on the effectiveness of the management of asthma in the working population. The importance of occupational factors in the onset of asthma in adults has been shown clearly in epidemiology studies, but the data obtained from workers from these studies remains fragmentary.

This French study was conducted in 2007 as a pilot study using a network of occupational physicians to collect information on asthma and AR and to evaluate the current level of asthma control and severity in the working population in France. The aim of the paper was to describe asthma control and severity using the GINA classification among salaried active workers according to the presence of current or previous AR comorbidity.

## Methods

### Study design

This was a cross-sectional epidemiological study. Conducted in two South West regions in France (Aquitaine and Midi-Pyrénées), the study relied on a network of voluntary occupational physicians (all sectors of activities and occupations). The occupational medical visit represents the opportunity to check the health status of employees. Occupational medicine is mandatory for all employees in France; consequently, every employee has regular compulsory medical examinations with an occupational physician.

Data collection took place over one year, beginning in September 2007. The study was conducted among all the employees of companies and establishments followed by the participating physicians in the two regions. The study protocol aimed for each volunteer physician to complete interviews for two employees a week, randomly selected among those they saw for regular periodic visits over 12 months. Each employee was selected using a random method (2 employees per week over 40 weeks for full-time occupational physicians), giving a total of 80 employees. The sample was extracted from the population of employees followed by the occupational physicians.

### Questionnaires

The first stage of data collection sought to identify all employees with asthma or respiratory symptoms. Each randomly selected employee completed a self-administered questionnaire, constructed from the questionnaires developed for the European Community Respiratory Health Survey (ECRHS) [15, 16]. It is composed of ten questions:

- “Have you had wheezing in your chest at any time in the last 12 months?”
- “Have you been woken by a shortness of breath at any time in the last 12 months?”
- “Have you had a shortness of breath after a physical effort in the last 12 months?”
- “Have you had a shortness of breath at rest in the last 12 months?”
- “Have you been woken by a coughing attack in the last 12 months?”
- “Have you been woken by a sense of suffocation in the last 12 months?”
- “Have you had an asthma attack in the last 12 months?”
- “Are you currently taking any medicine for asthma?”
- “Have you ever had asthma attacks?”
- “Do you have any nasal allergies including hay fever?”

Participants from the random sample were classified as “never had asthma” if the answers to all questions of the self-administered questionnaire were negative.

Each participating employee also filled out a questionnaire about their current job, including industry and occupation. Industry and occupation were coded according to the National Institute of Statistics and Economic Studies (INSEE) nomenclature 2008 [17, 18]. The industry and occupation groups were defined using the first level of these classifications. For employees reporting any respiratory symptoms (possible asthma) on the self-administered questionnaire, physicians filled in a medical questionnaire based on the ECRHS questionnaires [16, 19], including detailed information about the employee’s asthma (age at first and last attack, and frequency of symptoms during previous 3 months). Other variables included in the analysis were hospitalization, emergency visits due to respiratory disorders, drug treatment during the previous 12 months, smoking (nonsmokers, past smokers, and current smokers) and Body Mass Index (BMI).

### Description of the lung function measurements

We further had information on lung function values. The physician used a miniature electronic spirometer (PiKo-6®) to measure the respiratory function of each employee during the visit. Forced expiratory volume in one second (FEV1)/FEV6 ratios was measured three times and the mean FEV1/FEV6 ratio was calculated. Subjects were classified into three categories: Obstructive lung disease (≤70 %), borderline obstructive lung disease (71–79 %) and normal pulmonary function (≥80 %). The mean of the three measures of FEV / FEV6 was calculated as follows:

If all three measurements showed a difference of less than 20 % compared two by two, the mean of the three measurements was calculated.

If a measurement showed a difference of more than 20 % with the other two, it was excluded and the mean was calculated on the other two measurements.

If all three measures showed a difference of 20 % between them, the lowest measure was eliminated and the mean was calculated on the other two measures.

### Definition of current and previous asthma

In the self-administered questionnaire, subjects were categorized as “current asthmatics” (those who had experienced asthma attacks in the previous 12 months or who were currently taking asthma medication), “previous asthma” (those who gave a positive response to the question “Have you ever had asthma?” on the medical questionnaire but were not included in the current asthmatics) and “never had asthma” as previously described when all responses were negative.

### Definition of current and previous allergic rhinitis

AR was measured by the question “Do you have any nasal allergies including hay fever?” on the self-administered questionnaire. Participants were classified as “current allergic rhinitis” if they gave an affirmative response to this question on the self-administered questionnaire, and indicated antihistaminic treatment in the past 12 months on the medical questionnaire. Subjects were categorized as “previous allergic rhinitis” if they indicated that they had had AR in the self-administered questionnaire, but they had not taken antihistaminic treatment in the past 12 months. Otherwise, subjects were considered as “without AR”.

### Definition of respiratory symptoms

Subjects from the random sample were classified “respiratory symptoms but no asthma” if they had at least one positive response to the self-administered respiratory questionnaire except for questions “Have you had an asthma attack in the last 12 months?” and “Are you currently taking any medicine for asthma?” and gave a negative response to the question “Have you ever had asthma?” on the medical questionnaire.

### Asthma control

The classification of asthma control was also based on the GINA guidelines [7] according to the frequency of asthma symptoms during the previous 3 months. Asthma was considered to be controlled for subjects who had no daytime and nocturnal symptoms. Asthma was considered to be partly controlled if night-time asthma occurred < twice a month and daily symptoms < once a week. Asthma was considered to be uncontrolled if night-time asthma occurred > once a week and daily symptoms ≥ once a day.

The level of control for 39 current asthmatics (9 %) could not be assessed because they did not respond to questions on frequency of diurnal and nocturnal asthma symptoms.

### Asthma severity

**Fig. 1:**
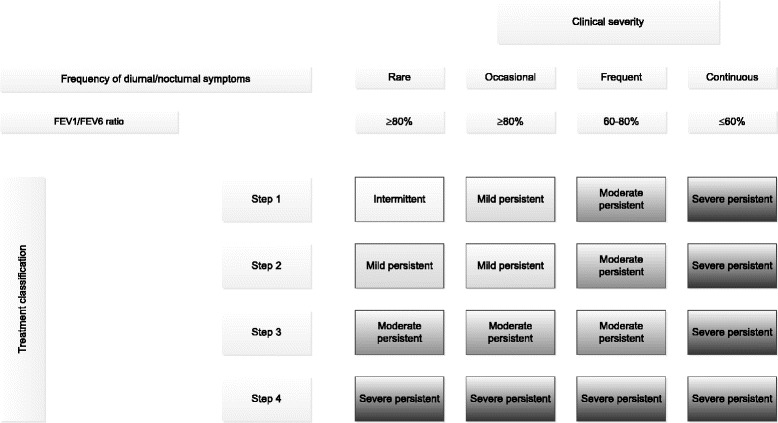
Classification of asthma severity based on the clinical severity and the treatment. Step 1: no medication or only “inhaled short-acting β2 agonists”; Step 2: inhaled corticosteroids daily or leukotriene modifier; Step 3: all subjects not allocated to the other groups; Step 4 (severe-persistent): oral corticosteroids daily or ≥3 short treatments in the previous 12 months or inhaled treatment combination of inhaled corticosteroids and inhaled long-acting β2 agonists

In agreement with the 2006 GINA guidelines [7], asthmatic subjects were classified according to severity based on a composite classification of clinical severity and daily medication regimen [6] (Fig. 1). Clinical severity was defined in four steps according to the frequency of diurnal and nocturnal symptoms in the previous 3 months and to pulmonary function, as measured by spirometry.

Treatment was classified in four steps according to reported medication use in the previous 12 months.

The final severity step was defined as the highest of the steps each patient was assigned to in the two independent classifications. Thus, each subject with asthma was classified as intermittent, mild-persistent, moderate-persistent and severe-persistent on the basis of the clinical and treatment classifications.

### Statistical analysis

Summary statistics are reported as percentages for categorical variables. Comparison of variables across strata was performed using the chi-square statistics. The level of significance was set at *P* < 0.05. Simple descriptive statistics were used to describe the study population and multivariate logistic regression analyses were performed to predict current asthma control based on various factors including AR (no, previous and current). Data analysis was carried out using Stata 9.2.

### Ethics

Authorization for data collection was obtained from the French data protection authority (agreement no. 907131 granted by the CNIL, Commission nationale de l’informatique et des libertés).

All subjects were informed about the objectives of the study, and those who agreed to participate filled out the questionnaires and underwent the measurements. Subjects were able to withdraw from the study at any time and were able to gain access to their personal data if required.

## Results

### Network of volunteer occupational physicians

Out of 846 occupational physicians in the two regions, 110 (13.0 %) agreed to participate in this pilot study. To assess the representativeness of the sample, we compared characteristics between the 736 not participating and the 110 participating physicians. Physicians from intercompany departments were overrepresented, while physicians from the other types of departments (autonomous, civil service, and social agricultural mutuality) were underrepresented. Overall, the mean number of employees included per physician was 63; only 37 % of the occupational physicians included the 80 employees initially planned.

### Sample characteristics

**Fig. 2:**
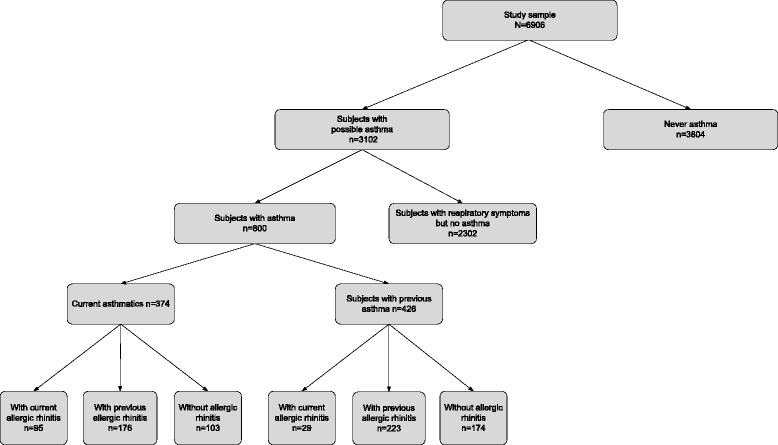
Study flow diagram

The study sample comprised 6906 workers (Fig. 2): 3839 in the Midi-Pyrénées and 3067 in Aquitaine, with a participation rate of 98.8 %. The mean age was 40 years (range 15–71) and 4080 (59.1 %) were male. The sex, age and industry for employees who refused to participate did not differ significantly from those who did take part. Overall, 3102 employees out of 6906 (44.9 %) were identified as having possible asthma (at least one positive response in the self-administered questionnaire). Eight hundred subjects (11.6 %) reported having had asthma at least once, and 374 (46.7 %) were current asthmatics. Of the current asthmatics, 271 (72.5 %) had AR, including 95 subjects with current AR.

### Characteristics of current asthmatics, subjects with previous asthma and subjects with respiratory symptoms but no asthma

The main characteristics of current asthmatics, previous asthma subjects and subjects with respiratory symptoms but no asthma are shown in Table 1. Current asthmatics were younger and were more likely to be underweight than subjects with previous asthma and subjects with respiratory symptoms but no asthma. Current asthmatics were significantly more likely to have been hospitalized during the last 12 months than subjects with previous asthma and subjects with respiratory symptoms but no asthma (respectively 17.2, 10.1 and 2.9 %; *P* < 0.001). The same trend was observed for emergency visits during the last 12 months (respectively 19.0, 4.9 and 3.2 %; *P* < 0.001).

**Table 1:** Characteristics of current and previous asthmatics and subjects with respiratory symptoms but no asthma

	Current asthmatics		Previous asthmatics		Subjects with respiratory symptoms but no asthma		
	*N* = 374		*N* = 426		*N* = 2302		
	n	%	n	%	n	%	*P* value
Sex							ns
Men	218	58.3	269	63.1	1307	56.8	
Women	156	41.7	157	36.9	995	43.2	
Age							<0.001
< 25 years	64	17.1	56	13.1	199	8.7	
25–34 years	98	26.2	137	32.2	558	24.2	
35–44 years	105	28.1	115	27.0	682	29.6	
45–54 years	83	22.2	93	21.8	650	28.2	
> 55 years	24	6.4	25	5.9	213	9.3	
Smoking habits							ns
Never	146	39.4	145	34.1	759	33.0	
Past	81	21.8	107	25.2	559	24.4	
Current	144	38.8	173	40.7	977	42.6	
Body Mass Index (kg/m2)							<0.001
Underweight (<18.5)	23	6.5	10	2.5	72	3.2	
Normal (18.5–25)	179	50.8	237	58.4	1221	55.2	
Overweight (25–30)	110	31.2	127	31.3	664	30.0	
Obese (>30)	40	11.4	32	7.9	255	11.5	
Hospitalization during the last 12 months	64	17.2	43	10.1	68	2.9	<0.001
Emergency visits during the last 12 months	71	19.0	21	4.9	74	3.2	<0.001
Occupational categories							ns
Managers	34	10.6	52	12.3	236	10.4	
Intermediate and supervisory	85	23.1	121	28.7	578	25.6	
Office and sales personnel	112	30.4	109	25.9	688	30.5	
Skilled laborers	75	20.4	77	18.3	501	22.2	
Unskilled laborers	57	15.5	62	14.7	256	11.3	
Industries							ns
Agriculture	3	0.8	3	0.7	33	1.4	
Manufacturing industries	70	18.9	89	21.0	515	22.5	
Construction industry	37	10.0	43	10.1	172	7.5	
Trade	50	13.5	55	13.0	310	13.6	
Transportation	13	3.5	13	3.1	64	2.8	
Personal service activities	15	4.0	21	5.0	104	4.5	
Real estate, business activities	66	17.8	85	20.0	399	17.4	
Finance	13	3.5	19	4.5	69	3.0	
Education, health, social	59	15.9	51	12.0	349	15.3	
Administration	44	11.9	45	10.6	271	11.8	

### Control and severity of current asthmatics

About 40 % of current asthmatics had moderate or severe persistent asthma. Among the 335 employees with current asthma who were classified according to the levels of control, 62.1 % were partly controlled or uncontrolled.

Subjects with partly controlled or uncontrolled asthma had higher rates of hospitalization compared with controlled asthma (24.0 and 8.7 %; *P* < 0.001) (Table 2). No differences appeared statistically significant according to sex, age, smoking habits, emergency visits, BMI, pulmonary function, AR, occupation or industry. Moderate or severe-persistent current asthmatics had a significantly higher rate of hospitalization at 23.7 % versus 10.7 % for intermittent or mild persistent current asthmatics (*P* < 0.001). Intermittent or mild persistent current asthmatics were more often smokers than moderate or severe-persistent current asthmatics (respectively 42.5 and 32.6 %; *P* < 0.001).

**Table 2:** Distribution of main sociodemographic and clinical characteristics according to current asthma control

	Control current asthma		Partly/no controlled current asthma		
	*N* = 127		*N* = 208		
	n	%	n	%	*P* value
Sex					ns
Men	81	63.8	119	57.2	
Women	46	36.2	89	42.8	
Age					ns
< 25 years	18	14.2	45	21.6	
25–34 years	42	33.1	53	25.5	
35–44 years	35	27.6	56	26.9	
45–54 years	24	18.9	44	21.1	
> 55 years	8	6.3	10	4.8	
Smoking habits					ns
Never	48	38.4	85	40.9	
Past	29	23.2	42	20.2	
Current	48	38.4	81	38.9	
Body Mass Index (kg/m2)					
Underweight (<18.5)	7	5.8	15	7.6	
Normal (18.5–25)	64	53.3	97	49.0	
Overweight (25–30)	38	31.7	63	31.8	
Obese (>30)	11	9.2	23	11.6	
Hospitalization during the last 12 months	11	8.7	50	24.0	<0.001
Emergency visits during the last 12 months	18	14.2	42	20.2	ns
Pulmonary function					ns
≤ 70 %	24	21.2	24	12.2	
71–79 %	40	35.4	76	38.8	
≥ 80 %	49	43.4	96	49.0	
Rhinitis allergic	92	72.4	152	75.0	ns
Occupational categories					ns
Managers	12	9.5	22	10.8	
Intermediate and supervisory	32	25.4	44	21.6	
Office and sales personnel	41	32.5	62	30.4	
Skilled laborers	22	17.5	44	21.6	
Unskilled laborers	19	15.1	32	15.7	
Industries					ns
Agriculture	-	-	-	-	
Manufacturing industries	23	18.4	36	17.5	
Construction industry	13	10.4	21	10.2	
Trade	20	16.0	28	13.6	
Transportation	6	4.8	6	2.9	
Personal service activities	6	4.8	7	3.4	
Real estate, business activities	19	15.2	43	20.9	
Finance	5	4.0	8	3.9	
Education, health, social	16	12.8	32	15.5	
Administration	16	12.8	24	11.6	

### Characteristics of current asthmatics with current or previous allergic rhinitis and without allergic rhinitis

The main characteristics of current asthmatics with current or previous AR and without AR are shown in Table 3. There was a higher proportion of females in the current asthmatics with current AR group compared to the previous AR and without AR groups (respectively 56.8, 39.7 and 38.8 %, *P* = 0.001). Current asthmatics with current and previous AR had lower rates of visits to the emergency physician compared to those without AR (respectively, 11.6, 17.1 and 29.1 %, *P* < 0.001). Different patterns of occupational categories were observed across patient groups (*P* < 0.001), with asthmatics with current AR being overrepresented in the manager, intermediate and supervisory personnel and office and sales personnel categories compared to the other groups.

**Table 3:** Characteristics of current asthmatics with current and previous allergic rhinitis (AR) and without allergic rhinitis

	Current asthmatics with current AR		Current asthmatics with previous AR		Current asthmatics without AR		
	(*N* = 95)		(*N* = 176)		(*N* = 103)		
	n	%	n	%	n	%	*P* value
Sex							<0.001
Men	41	43.2	114	52.3	63	61.2	
Women	54	56.8	62	39.7	40	38.8	
Age							ns
< 25 years	14	14.7	31	17.6	19	18.4	
25–34 years	25	26.3	51	29.0	22	21.4	
35–44 years	28	29.5	47	26.7	30	29.1	
45–54 years	21	22.1	37	21.0	25	24.3	
> 55 years	7	7.4	10	5.7	7	6.8	
Smoking habits							ns
Never	43	45.7	68	39.1	35	34.0	
Past	17	18.1	43	24.7	21	20.4	
Current	34	36.2	63	36.2	47	45.6	
Body Mass Index (kg/m2)							ns
Underweight (<18.5)	6	7.1	9	5.3	8	8.2	
Normal (18.5–25)	42	49.4	88	51.8	49	50.5	
Overweight (25–30)	27	31.8	54	31.8	29	29.9	
Obese (>30)	10	11.8	19	11.2	11	11.3	
Hospitalization during the last 12 months	18	18.9	27	15.4	19	18.4	ns
Emergency visits during the last 12 months	11	11.6	30	17.1	30	29.1	<0.001
Pulmonary function							ns
≤ 70 %	9	1.9	27	16.9	17	17.9	
71–79 %	37	42.0	56	35.0	36	37.9	
≥ 80 %	42	51.1	77	48.1	42	44.2	
Occupational categories							<0.001
Managers	14	15.2	17	9.7	8	7.9	
Intermediate and supervisory	33	35.9	47	26.9	32	31.7	
Office and sales personnel	28	30.4	41	23.4	16	15.8	
Skilled laborers	7	7.6	42	24.0	26	25.7	
Unskilled laborers	10	10.9	28	16.0	19	18.8	
Industries							ns
Agriculture	-	-	-	-	-	-	
Manufacturing industries	12	13.0	39	22.3	19	18.4	
Construction industry	8	8.7	21	12.0	8	7.8	
Trade	9	9.8	26	14.9	15	14.6	
Transportation	-	-	-	-	-	-	
Personal service activities	-	-	-	-	-	-	
Real estate, business activities	13	14.1	30	17.1	23	22.3	
Finance	-	-	-	-	-	-	
Education, health, social	21	22.8	22	12.6	16	15.5	
Administration	14	15.2	20	11.4	10	9.7	

No differences in rates of nocturnal and diurnal symptoms between the three groups were observed (Table 4). More current asthmatics with current AR had at least one asthma attack in the previous year compared to current asthmatics without AR (respectively 77.9, 62.5 and 60.2 %; *P* < 0.001). Nearly 40 % of subjects with current AR had moderate or severe-persistent asthma. In 68.8 % of cases with current asthma and AR, asthma was only partly controlled or uncontrolled. No differences in terms of control and severity were observed between current asthmatics with or without AR.

**Table 4:** Clinical characteristics, control and severity in current asthmatics with and without allergic rhinitis (AR)

	Current asthmatics with current AR		Current asthmatics with previous AR		Current asthmatics without AR		
	(*N* = 95)		(*N* = 176)		(*N* = 103)		
	n	%	n	%	n	%	*P* value
Nocturnal symptoms in the previous 3 months							ns
No symptoms or <2 times a month	76	83.5	132	82.5	74	84.1	
≥ 2 times a month but < once a week	8	8.8	16	10.0	5	5.7	
> once a week	7	7.7	12	7.5	9	10.2	
Diurnal symptoms in the previous 3 months							ns
No symptoms or < once a week	60	65.9	122	76.2	63	71.6	
≥ once a week but < once a day	23	25.3	28	17.5	18	20.4	
≥ once a day	8	8.8	10	6.3	7	8.0	
At least one asthma attack in the previous year	74	77.9	110	62.5	53	60.2	<0.001
Absenteeism in the previous 3 months	4	4.5	6	4.0	7	8.5	ns
Severity							ns
Intermittent	13	13.7	40	22.7	18	17.5	
Mild-persistent	42	44.2	76	43.2	46	44.7	
Moderate	10	10.5	12	6.8	12	11.6	
Severe-persistent	30	31.6	48	27.3	27	26.2	
Control							ns
Controlled	28	31.2	64	40.5	35	40.2	
Partly controlled	49	54.4	76	48.1	40	46.0	
Uncontrolled	13	14.4	18	11.4	12	13.8	

More than half of the subjects with poor asthma control did not use effective medication to reduce airway inflammation. Indeed, 51.6 % current asthmatics with current AR using 1–2 step treatment (Fig. 1) were observed among partly controlled/uncontrolled asthmatics. About 60 % of partially controlled/uncontrolled asthmatics had a 1–2 step treatment for current asthmatics with previous AR and current asthmatics without AR.

Multiple logistic regression showed that compared to those with no AR, the odds of partly or no control asthma were 40 % higher among those with current AR, though this was not statistically significant (adjusted OR, 1.4; 95 % CI, 0.8–2.7) (Table 5). There were no differences between current asthmatics with intermittent or mild-persistent severity and current asthmatics with moderate or persistent severity in terms of presence of AR.

**Table 5:** Logistic regression for current asthma control based on presence of allergic rhinitis for employees

	Odds ratio [95%CI]^a^
Allergic rhinitis	
No	ref
Previous	1.0 [0.6–1.7]
Current	1.4 [0.8–2.7]

^a^Logistic model adjusted for age, sex, hospitalization and smoking status

### Characteristics of previous asthmatics according to current or previous allergic rhinitis

A statistically significant difference was found between smoking habits across the three groups (previous asthmatics with current and previous AR and previous asthmatics without AR), with the lowest percentage of current smokers among previous asthmatics with current AR (respectively, 13.8, 38.3 and 48.3 %, *P* = 0.002). No statistically significant differences according to occupational category and industry were found between previous asthmatics with current and previous AR and previous asthmatics without AR.

## Discussion

Similar to epidemiological data in the general population [20], our findings suggest that self-reported AR is present in many current asthmatics in a population of salaried employees in France. In this study of salaried workers, about two thirds of subjects with asthma suffered from AR. Most current asthmatics with or without AR had uncontrolled asthma with inappropriate treatment. Moreover, current asthmatics with AR have a significantly lower risk of subsequent asthma-related events (such as emergency department visits) than current asthmatics without AR. In contrast, they had more asthma attacks. In our study, no significant differences were observed between current asthmatics with or without AR in terms of control and severity.

We observed the same rate of uncontrolled asthma in the current asthmatics with or without AR. The odds of partly or no control asthma were 40 % higher among those with current AR although not statistically significant whereas clinically diagnosed AR has been found to be associated with significantly worse asthma control [13, 14, 21]. Clatworthy et al. suggested that having comorbid AR is a marker for the presence of more difficult-to-control asthma [13]. They reported that the degree of rhinitis was important with those having severe rhinitis exhibiting the worst asthma control, followed by those having mild rhinitis and then those having no rhinitis symptoms. However, in our study, we did not evaluate the severity of AR.

In agreement with other studies, we found that about 40 % of current asthmatics with or without AR could be affected by moderate-to-severe asthma [22, 23]. Furthermore, our findings confirm the existence of a high proportion of current asthmatics with inadequate asthma control. Previous studies found similar results, despite possible differences in the definition of asthma control. For example, the Asthma Insights and Reality in Europe (AIRE) study, which analyzed 2050 asthmatic patients from different European countries, found that only 35 % of them had totally controlled asthma [24]. Even with seemingly appropriate and effective therapy, fewer than half of patients have good control of their disease [25].

About half of participating employees with poor asthma control did not use effective medication to reduce airway inflammation. In Italy, the lack of treatment for asthma has been mooted as one of the main reasons for poor control asthma [26]. This was not the case in our study, in which the vast majority of current asthmatics with or without AR were receiving treatment. Nevertheless, treatment was self-reported so we cannot rule out the possibility that some subjects did not actively follow the treatment prescribed. Comparison with previous studies showed that asthmatics in France, as elsewhere, have considerably increased their use of asthma drugs in the last decade, while the control of symptoms has worsened [27, 28]. While this increase in drug use is insufficient to control the disease, it is likely that other dimensions such as inadequate patient education or treatment that is not adapted to the severity level are the main reasons for the poor asthma control. For uncontrolled asthmatics with suboptimal treatment, there are other possible explanations, such as occupational exposures playing a role in both the development and exacerbation of asthma. Le Moual et al. suggested a progressive increase in lack of asthma control among persons currently exposed to asthmogens at work [29]. They underlined the necessity for physicians to consider occupational exposures as a risk factor for asthma.

By concentrating on the patient’s workplace, the clinician has an opportunity to practice preventive medicine: to recognize substances in the patient’s micro- and macro-environment that are causing the problems and to intervene by altering the environment or removing the patient from the environment.

The presence of AR often precedes the onset of asthma and increases morbidity, therapeutic needs and use of healthcare resources in patients with asthma [30]. One study reported that adult asthmatics with comorbid AR consumed more asthma-related healthcare resources in terms of general practitioner visits, hospitalizations and prescription medication costs than patients with asthma alone [31]. In our study, the presence of self-reported previous or current AR in subjects with current asthma resulted in less emergency visits compared with current asthmatics without AR, but not in fewer hospitalizations. These results may be explained by the fact that treating comorbid AR is associated with reductions in emergency visits and confers better asthma-related outcomes [32]. Reducing asthma symptoms with therapy for AR could potentially decrease asthma-related health care resource utilization. Some studies have demonstrated that among patients with asthma and comorbid AR, those receiving treatment for AR have a significantly lower risk of subsequent asthma-related events than those who were not treated [32, 33]. Treatment of AR simultaneously produces a favorable effect on symptoms of asthma and concurrent improvement in lung function and bronchial hyperresponsiveness. Moreover, this study suggests that the presence of AR in current asthmatics results in a higher rate of asthma attacks suggesting that these subjects have received more treatment, reducing the risk of an Emergency Department visit for asthma.

In this article, the relationship between asthma and occupational characteristics has not been discussed in more detail. An article on asthma prevalence according to activities and occupations of the employees sampled by our team will be published.

### Strengths and limitations

To our knowledge, this is one of the first studies to evaluate the impact of AR on asthma in a working population. In this study, employees with possible asthma received lung function assessment with spirometry, whereas most of the previous studies using databases did not use spirometry, which is the recommended method. The use of expiratory flow measurements by physicians is infrequent in both general and occupational practice. This study calls for greater awareness of the need for asthma management according to GINA guidelines. It is important that physicians use such tools given that asthma can be caused by workplace exposure. The validity of the FEV1/FEV6 ratio has been examined and seems to be a valid tool for use in the general population [34].

However, our study has some limitations. It was a cross-sectional study and so it was not possible to establish time trends between the determinants studied and asthma severity and control. It was based on the voluntary participation of occupational physicians. Although 13 % of all occupational physicians participated, this was not sufficient to identify a large enough number of asthmatics enabling more robust results.

In our study, subject inclusion was based on a random selection among employees undergoing compulsory periodic occupational health visits. Other occupational visits were not taken into account (pre-employment medical examinations, medical examinations on return to work and on-demand consultations) and subjects with asthma may thus have been missed. In addition, this study does not take into account self-employed workers and workers not currently in labor market (unemployment or disability which may include subjects with respiratory disorders). Further selection bias is possible in that vulnerable workers may have self-selected themselves out of the industries where exposure worsens their asthmatic symptoms. This healthy worker effect is a common problem in all cross- sectional epidemiological studies. It results from the phenomenon that asthma or related symptoms may influence job choice through the avoidance of potentially hazardous occupational exposure through an initial or an ongoing selection process [35].

## Conclusions

Most current asthmatics both with and without AR had uncontrolled asthma, with inappropriate treatment. The presence of comorbid AR in asthmatic employees resulted in a lower rate of emergency visits compared with employees with asthma alone which might be due to treatment. Thus, it would seem appropriate to identify AR in asthmatic subjects, especially for occupational physicians given the substantial risk of asthma exacerbation in the workplace. However, future work is needed to confirm these findings and the relation between occupational exposure and poor asthma control may warrant further study.

## Abbreviations

AR: Allergic rhinitis
ECRHS: European Community Respiratory Health Survey
GINA: Global Initiative for Asthma
INSEE: National Institute of Statistics and Economic Studies
FEV: Forced expiratory volume.
